# Application of peptides with an affinity for phospholipid membranes during the automated purification of extracellular vesicles

**DOI:** 10.1038/s41598-020-75561-0

**Published:** 2020-10-30

**Authors:** Takenori Ishida, Takuma Hashimoto, Kanako Masaki, Hisakage Funabashi, Ryuichi Hirota, Takeshi Ikeda, Hideji Tajima, Akio Kuroda

**Affiliations:** 1 Unit of Biotechnology, Graduate School of Integrated Sciences for Life, Hiroshima University, 1-3-1 Kagamiyama, Higashi-Hiroshima, Hiroshima 739-8530 Japan; 2 Precision System Science Co., Ltd., 88 Kamihongo, Matsudo, Chiba 271-0064 Japan

**Keywords:** Biological techniques, Biotechnology, Biomarkers

## Abstract

Extracellular vesicles (EVs), such as exosomes, have garnered increasing interest because of their potential clinical applications that range from diagnostics to therapeutics. The development of an automated and reproducible EV purification platform would therefore aid the introduction of EV biomarkers and therapies into the clinic. Here, we demonstrate that K8- as well as K-16 peptides (containing 8 and 16 lysine residues with dissociation constants of 102 nM and 11.6 nM for phosphatidylserine, respectively) immobilized on magnetic beads can capture small EVs (< 0.2 µm) from culture supernatants of MCF7 human breast cancer cells. Importantly, the bound EVs could be dissociated from the beads under mild conditions (e.g. 0.5 M NaCl), and the isolated EVs had the typical shapes of EVs under SEM and TEM with a mean particle size of 99 nm. Using the peptide-immobilized beads, we adapted a pre-existing bench top instrument for magnetic separation to perform automated EV purification with higher purity and yield than that obtained using the standard ultracentrifugation method.

## Introduction

Extracellular vesicles (EVs) can be divided into three groups based on their biogenesis: exosomes, microvesicles (MVs), and apoptotic bodies^1^. Exosomes are a particular type of EV surrounded by a phospholipid bilayer (approximately 50–100 nm in size) that are formed inside endosomal compartments. In contrast, MVs, which are also surrounded by a phospholipid bilayer and generally 100–1000 nm in size, are formed by budding from the plasma membrane^2^. Apoptotic bodies range from 1 to 5 µm^2^. In this report, the term EVs will refer to exosomes and MVs smaller than 0.2 µm only. Current research in the field is primarily focused on exosomes containing proteins and micro RNAs (miRNAs). As these exosome-enclosed proteins and miRNAs are protected from digestion in the blood, exosomes can provide crucial information on the status of multiple diseases and medical conditions^3^. In this respect, a variety of exosomal proteins and miRNAs have been identified as potential diagnostic, prognostic, or therapeutic biomarkers^4–9^. In addition, numerous studies have shown that exosomes have a wide range of biological functions, such as cell–cell communication and signalling, and thus may mediate their therapeutic effects by transmitting genetic and non-genetic information between cells. For example, mesenchymal stem cell (MSC)-derived EVs, including exosomes, can recapitulate the therapeutic and regenerative effects of MSCs on damaged tissues/organs in models of myocardial ischemia^10^, acute tubular injury^11^, stroke^12^, acute lung injury/ischemia^13,14^, and skin wounds^15,16^. EVs therefore have a great potential in various clinical applications ranging from diagnostics to therapeutics.

To date, several techniques for the isolation of EVs have been developed, including those based on differential ultracentrifugation, size, immunoaffinity capture, and microfluids, as well as EV precipitation techniques^17,18^. Among these, differential ultracentrifugation-based techniques are considered the gold standard and the most commonly used techniques for EV isolation. However, the EVs isolated using these techniques often contain external proteins and lipoproteins, resulting in a lower purity of the isolated EVs. Moreover, this method is time consuming, labor intensive, and therefore not suitable for high throughput diagnostics. Furthermore, the collapse and damage of EV membranes, or EV aggregation, following isolation by differential ultracentrifugation have been reported^19^. In particular, the isolation of structurally as well as biologically intact EVs is required for their therapeutic application. It has been noted that size-exclusion chromatography can potentially yield highly purified EVs because it is typically performed using gravity flow, so that the vesicle structure and integrity largely remain intact and the biological activity of EVs is preserved. However, this approach is limited by a lack of scalability, a feature required for therapeutic applications. Although a series of commercial isolation kits have been developed to improve yield, the purity of the EVs extracted by these kits is compromised. Significant effort is still required to develop better methods for the isolation and purification of EVs.

Separation techniques using magnetic beads, in which the surface is modified with an affinity ligand, have several advantages compared with other separation procedures. Magnetic separation is usually very gentle on the target molecules as long as they can be easily released from the magnetic particles under mild conditions. The efficiency of magnetic separation procedures is especially useful in large-scale or high throughput operations. Magnetic separation techniques have often been used as a basis for various automated procedures. For example, DNA purification using magnetic particles has been used for the automated extraction and determination of DNA molecules from cells, blood, and organs.

High-affinity antibody-antigen complexes are generally difficult to dissociate, and often require extreme pH conditions or denatured conditions for dissociation. Thus, antibodies against EV-surface markers, such as anti-CD9 and anti-CD63 antibodies, cannot serve as suitable affinity ligands for the isolation of intact EVs. Recently, Tim4, which binds to phosphatidylserine in EV membranes in a Ca^2+^-dependent manner—and can be dissociated in the presence of EDTA—has been used as the affinity ligand for EV purification^20^. Peptides with binding affinities sufficient to capture target molecules without undue losses but which are still capable of allowing the elution of bound molecules under mild conditions have also been identified^21–23^.

Owing to their synthetic nature and small size, peptides have excellent chemical stability, and therefore peptide-based affinity matrices are more robust and able to endure the extreme pH conditions during regeneration steps than protein-based matrices. Furthermore, peptides can be produced under Good Manufacturing Practice conditions in bulk and at a low cost^23^; additionally, site-directed immobilization with a high ligand density can be easily achieved with peptides. Cell-penetrating peptides are generally arginine- and lysine-rich, and can bind to cell membranes^24,25^. The initial membrane association is mediated by the interaction of the arginine and lysine side chains with negatively-charged groups on the cell surface. Molecular dynamics simulations have shown that both octa-lysine and octa-arginine can bind to lipid bilayer surfaces^26^. However, whether these peptides can be used as affinity matrices of EVs is unclear.

A selective, reproducible, robust, and high throughput technique for the isolation/purification of intact EVs is critical to meet the demands of increasing EV research, for surveying EV biomarkers, and developing EV therapeutic applications^17^. We believe that the automation of EV purification is essential for the development of reproducible and high throughput methods for the isolation of intact EVs. However, there have been no reports describing the development of automated EV purification platforms. Here, to develop such a platform, we first tested lipid-binding peptides with different numbers of lysine residues that could be used for the magnetic separation of EVs. Following optimization of the number of lysine residues in the peptides, we explored a range of different elution conditions that allowed for the release of EVs from the peptides. Finally, we used the peptide-immobilized magnetic beads to develop an automated EV purification system.

## Results

### A lysine-rich affinity peptide for EV capture

**Figure 1:**
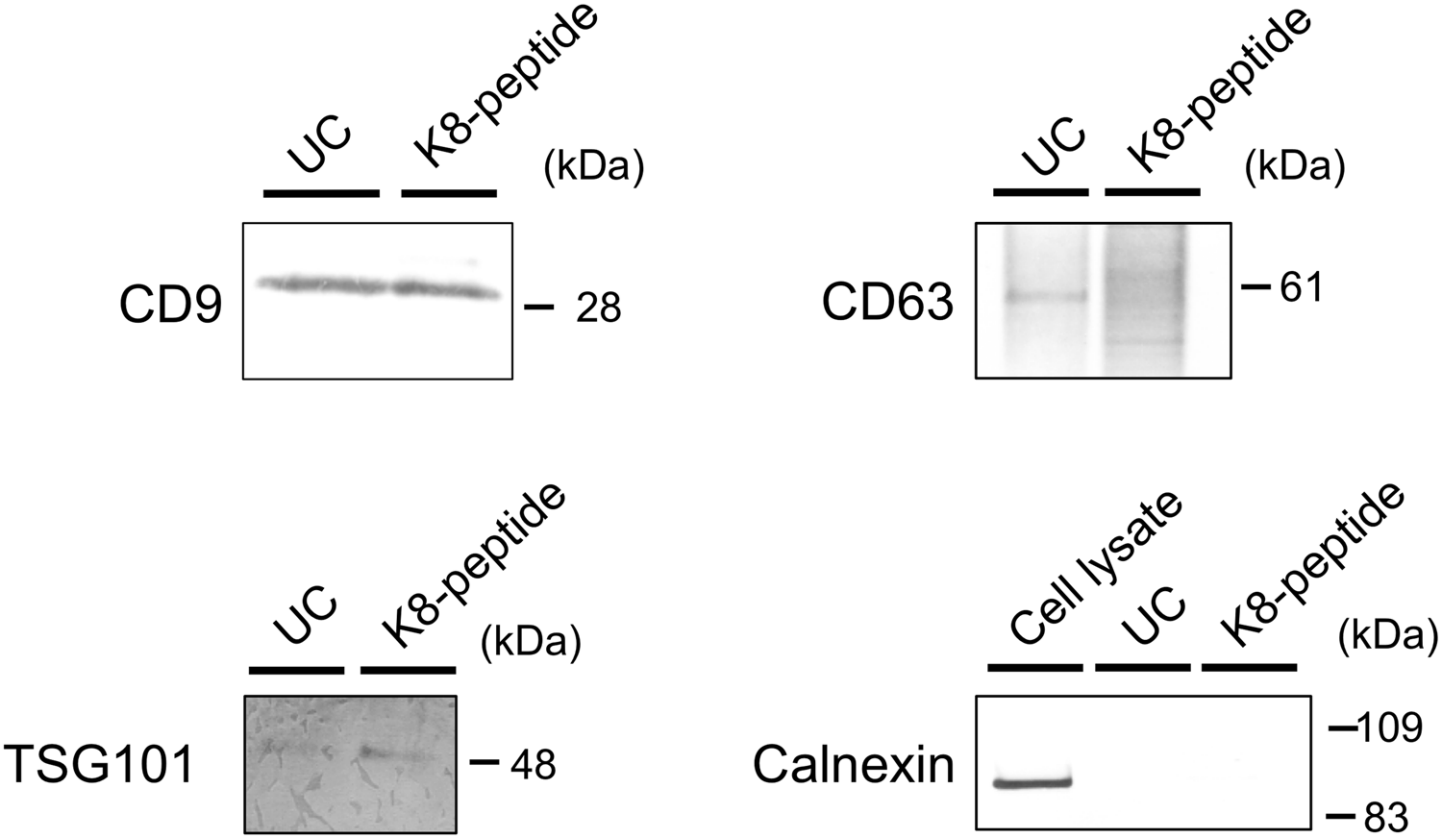
Western blotting for the EVs bound to K8-peptide magnetic beads. One milliliter of culture supernatant from MCF7 cells was separated by ultracentrifugation (UC), or mixed with K8-peptide beads, and the bound EVs were extracted with SDS buffer. Western blotting for this material was performed using anti-human CD9, CD63, TSG101, and Calnexin antibodies. For western blots using the Calnexin antibody, a cell lysate was also analyzed. Images of the full western blots are shown in Supplementary Figs. S1–S4.

Lysine- and arginine-rich peptides have an affinity for phospholipid bilayers^24,25^. Thus, we tested whether lysine-rich peptides can be used as affinity peptides that bind to EV membranes. A biotinylated peptide comprising three GGGS linkers and eight lysine residues (K8-peptide) was conjugated to streptavidin-immobilized magnetic beads and then mixed with a culture supernatant derived from MCF7 cells. After mixing, to examine whether EVs could bind to the peptide-conjugated beads, the beads were recovered using a magnetic stand and then washed. Following this step, proteins extracted from the beads with sodium dodecyl sulfate (SDS)-sample buffer were subjected to western blotting by using anti-CD9 and anti-CD63 antibodies (Fig. 1). Material bound to the K8-peptide, as well as an EV fraction obtained using the classical differential ultracentrifugation methods, was found to contain CD9 and CD63 EV marker proteins. The bound material also contained another marker protein TSG101, but not the endoplasmic reticulum protein Calnexin (negative control) (Fig. 1). These results suggested that the K8-peptide could capture EVs from culture supernatants.

**Figure 2:**
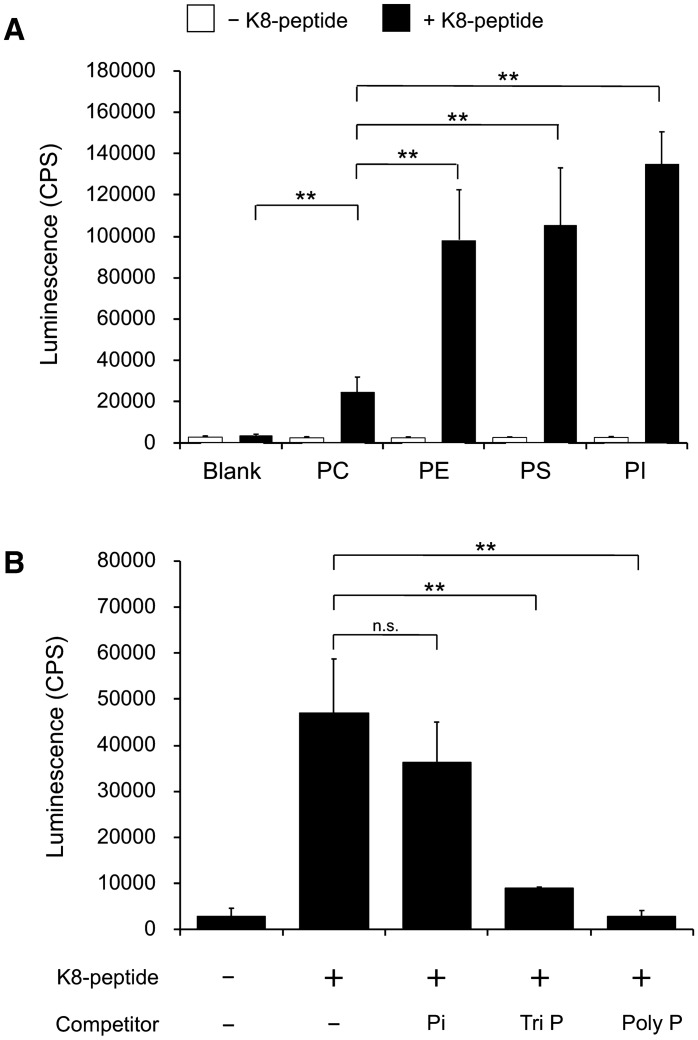
Phospholipid binding by the K8-peptide. (**A**) The biotinylated K8-peptide was added to the original plate (blank) and phosphatidylcholine (PC)-, phosphatidylethanolamine (PE)-, phosphatidylserine (PS)-, phosphatidylinositol (PI)-immobilized plate wells. The amount of K8-peptide bound to the wells was measured using HRP-conjugated streptavidin. Luminescence was also measured in the absence of K8-peptide (open box). (**B**) A competition assay was performed using the biotinylated K8-peptide and the PS-immobilized plate in the presence of 30 mM phosphate (Pi), 10 mM triphosphate (Tri P), or 2 mM polyphosphate (15 phosphate residues) (Poly P). The *p*-values were calculated using Student’s *t* tests (**p < 0.01; *n.s.* not significant, *p* ≥ 0.05). Each experiment was performed with three technical replicates.

To analyze the affinity of the K8-peptide for EV membranes, phosphatidylcholine (PC)-, phosphatidylethanolamine (PE)-, phosphatidylserine (PS)-, and phosphatidylinositol (PI)-immobilized plates were used in a binding assay. The K8-peptide bound strongly to PE, PS, and PI, but relatively weakly to PC (Fig. 2A). The lipid contents in exosomes from several kinds of cells have been summarized by Skotland et al.^27,28^. Although the phospholipid of EVs derived from different cell types appeared to vary significantly, the PI content was found to be low relative to other phospholipids^27^. Since the affinity of K8-peptide for PC is relatively low, we assume that the interaction of the K8-peptide with PS and PE mainly contributes to its affinity for EV membranes.

The affinity of a lysine-rich peptide for a lipid membrane can be attributed to its ability to form hydrogen bonds with the lipid phosphate group^29^. Molecular simulation has shown that a Tat peptide containing two lysine and six arginine residues—known to be a cell-penetrating peptide—sequesters phosphate groups from neighbouring phospholipids^30^. To elucidate the binding mechanism of the K8-peptide, a competition assay was performed using phosphate-containing competitor compounds. The binding of the K8-peptide to PS was inhibited by 10 mM tri-polyphosphate, but not significantly by 30 mM phosphate (Fig. 2B). However, the binding was completely inhibited by 2 mM polyphosphate (15 phosphate residues) (Fig. 2B). The strong inhibition by polyphosphate suggests that the K8 peptide–lipid complex is stabilized through binding to several phosphate groups in neighbouring phospholipid molecules.

### Effect of the number of lysine residues in the affinity peptide on EV capture

**Figure 3:**
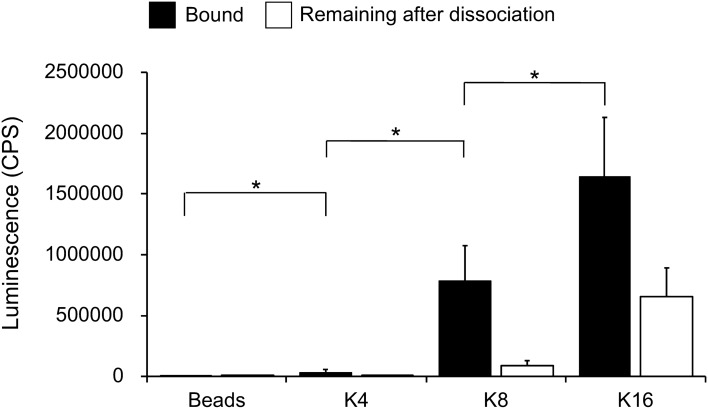
Effect of the number of lysine residues in the affinity peptides on EV capture. The amount of EVs bound to K4-, K8-, and K16-peptide-immobilized magnetic beads were analyzed using an anti-CD9 antibody (closed box). After the dissociation of the EVs using 0.5 M NaCl, the material that remained on the beads was analyzed using an anti-CD9 antibody (open box). The *p*-values were calculated using Student’s *t*-tests (*p < 0.05; *n.s.* not significant, *p* ≥ 0.05). Each experiment was performed with three technical replicates.

To analyze the effect of the number of lysine residues in the affinity peptide on EV capture, biotinylated peptides comprising 4 lysine residues (K4-peptide) or 16 lysine residues (K16-peptide) with GGGS linkers were synthesized and conjugated to streptavidin-immobilized magnetic beads. The K4-, K8-, and K16-peptide-immobilized beads were mixed with MCF7 culture supernatants and the levels of CD9 bound to the K4-, K8-, and K16-peptide-immobilized beads were measured. The levels of CD9 bound to the K4-peptide-immobilized beads were slightly above those found for beads only, whereas the levels on the K8- and K16-peptide-immobilized beads far exceeded those seen with beads only (Fig. 3, bound), suggesting that peptides with more than eight lysine residues are required for efficient EV capture.

**Figure 4:**
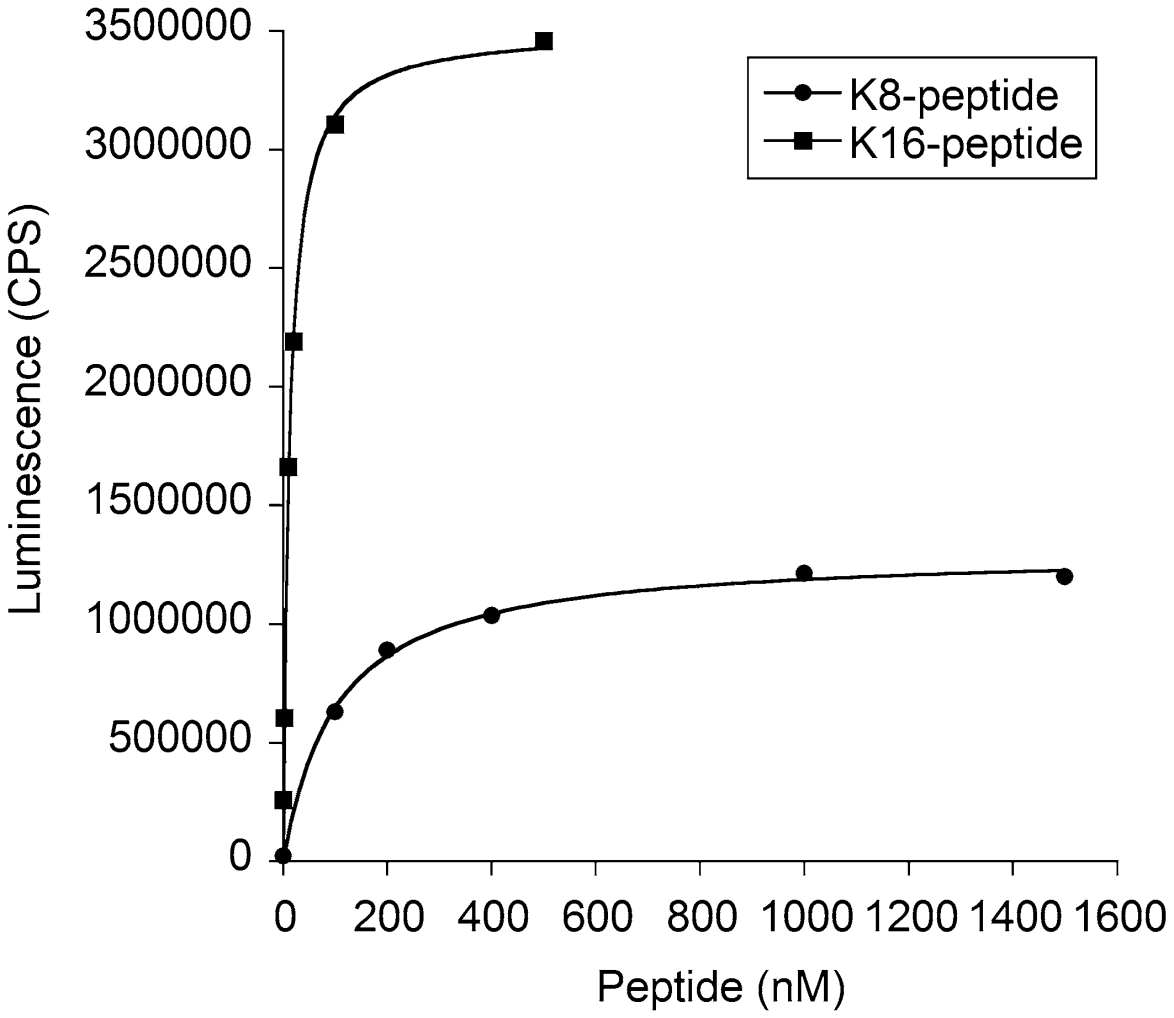
Dissociation constants for the binding of K8- and K16-peptides to PS. The binding experiment was performed as described in Fig. 2 and “Methods”. The resulting data were fitted to a Langmuir isotherm to determine the dissociation constants (Kds) and the maximum binding capacities (Bmaxs) for the binding of K8- and K16-peptides to phosphatidylserine (PS).

To further characterize the peptide affinity for EV membranes, the dissociation constants (Kds) of the K8- and K16-peptides for PS were measured. The Kds of the K8- and K16-peptides for plate-immobilized PS were 102 and 11.6 nM, respectively (Fig. 4). The maximum binding capacity (Bmax) of the K16-peptide to PS-plates was approximately 2.7-fold that of the K8-peptide. This higher affinity of the K16-peptide for PS could explain why the K16-peptide-immobilized beads captured EVs more efficiently than the K8-peptide-immobilized beads (Fig. 3). However, as described below, the efficiency of EV dissociation from the K16-peptide-immobilized beads was lower than that obtained for the K8-peptide-immobilized beads, indicating there is an optimum number of lysine residues required for efficient EV isolation.

### Conditions for the dissociation of EVs from peptide-immobilized magnetic beads

**Figure 5:**
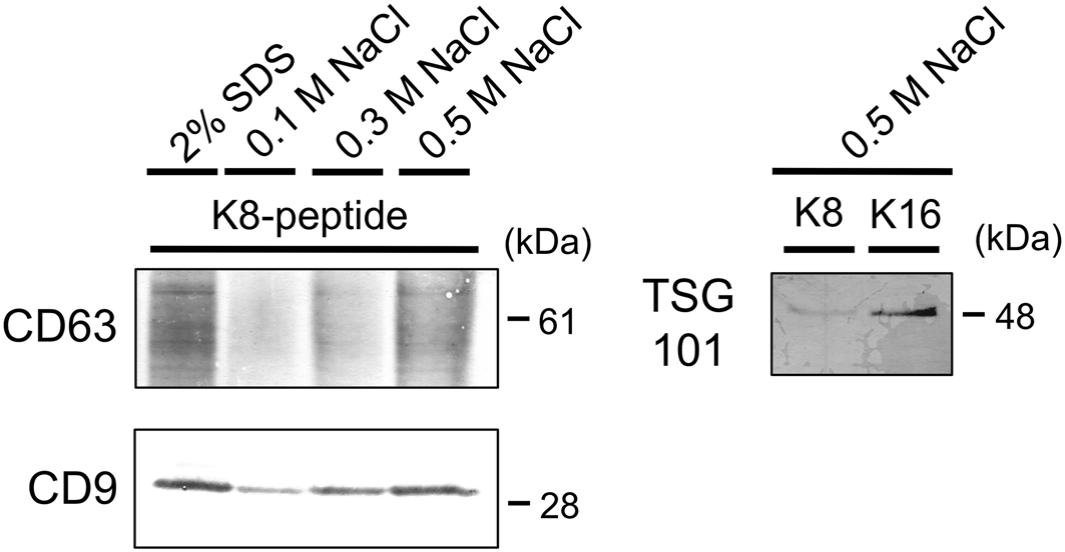
Conditions used for the dissociation of EVs from K8-peptide-immobilized magnetic beads. EVs bound to K8-peptide-immobilized magnetic beads were dissociated with SDS sample buffer or buffers with the indicated concentrations of NaCl. The released materials were analyzed by western blotting using anti-CD9 and CD63 antibodies. The materials released with 0.5 M NaCl were analyzed by western blotting using an anti-TSG101 antibody. Images of the full western blots are presented in Supplementary Fig. S3 (for TSG101), Fig. S5 (for CD9), and Fig. S6 (for CD63).

To purify intact EVs, we explored mild conditions under which EVs could be dissociated from the peptide-immobilized magnetic beads. Because lysine can function as a competitive ligand in the binding of the K8-peptide to the membrane, we first tried to dissociate the EVs from the K8-peptide using a buffer containing 0.5 M lysine. Unfortunately, the efficiency of dissociation was not satisfactory (less than 40%). However, we found that buffers containing 0.5 M NaCl were able to elute most of the CD9 and CD63 (Fig. 5). The intravesicular marker protein TSG101 was also detected in the fractions eluted from the K8-peptide and K16-peptide-immobilized beads by 0.5 M NaCl (Fig. 5). Buffers containing 0.5 M KCl as well as 0.3 M MgCl_2_ were also effective for the dissociation of EVs from the K8-peptide-immobilized beads (almost 100% and 90%, respectively).

To examine whether EVs bound to the K16-peptide-immobilized beads were dissociated in the presence of 0.5 M NaCl, the amount of CD9 remaining on the K16-peptide-immobilized beads after dissociation was measured (Fig. 3, remaining after dissociation). The efficiency of the dissociation of EVs from the K16-peptide-immobilized beads was less than their efficiency of dissociation from the K8-peptide-immobilized beads. The difference between “bound” and “remaining after dissociation”, representing the isolation yield, was not significantly different for the K8- and K16-peptide-immobilized beads (Student’s *t* test, n = 3 preparations, *p* < 0.05). Thus, although longer lysine peptides could capture the EVs more efficiently, their dissociation efficiency under mild conditions was lower.

### Purification of EVs using affinity peptides

**Figure 6:**
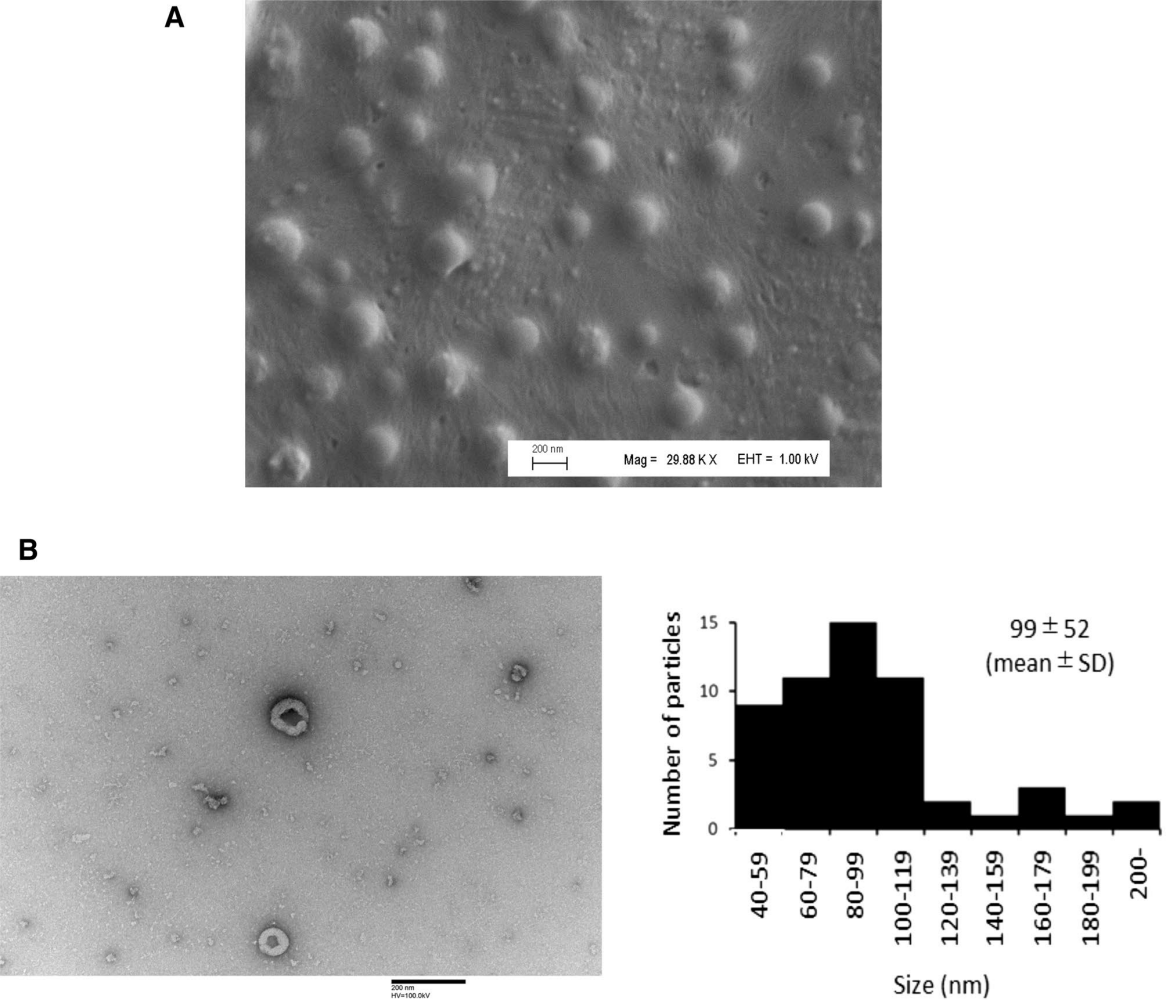
Electron microscopy of the EVs dissociated from K8-peptide-immobilized magnetic beads. (**A**) Purified EVs were visualized under SEM (Magnification: × 29,880; Scale bar 200 nm). (**B**) Purified EVs were visualized under TEM (Magnification: × 100,000; Scale bar 200 nm). The size distribution of EVs in 42 fields of view is indicated (right).

We observed the EVs dissociated from the magnetic beads on a 0.5 M NaCl buffer by electron microscopy. Most of the EVs isolated using the K8-peptide-immobilized beads had the typical round shape of EVs under SEM (Fig. 6A) and a cup- or saucer-like shape under TEM^31^ with a mean particle size of 99 nm (Fig. 6B). The latter observation may reflect the collapse of the EVs as a result of the dehydration process during sample preparation^31^. These results suggested that EV capture and dissociation using the affinity peptide-immobilized magnetic beads could be used to purify intact EVs. To assess the purity of the EVs, the EV amount was measured using a CD9/CD63 sandwich ELISA kit, and normalized to a CD9/CD63 fusion protein. The purity of EVs was expressed as the amount of CD9/CD63 fusion protein/µg total protein. The purity and yield of EVs manually isolated using the K8-peptide-immobilized beads were 43-fold and 2-fold higher than that observed using the differential ultracentrifugation method (Table 1), respectively. When K16-peptide-immobilized magnetic beads were used, the yield was increased to 52%, but the purity was decreased approximately 2-fold (Table 1), suggesting a trade-off between purity and yield; thus, K8- or K16-peptides should be used when purity or yield, respectively, are the priority.

**Table 1:** Comparison of EV purification yields from culture supernatants of MCF7 cells using different techniques.

Sample	Purification method	CD9/CD63 amount in EVs (pg)	Total protein (μg)	Purity (CD9/CD63 amount/total protein)	Purification fold	Yield (%)
MCF7 supernatant		312 ± 8^a^	1344 ± 38^a^	0.233 ± 0.005^a^	1^a^	100^a^
	Differential ultracentrifugation	23 ± 6	4.54 ± 0.09	4.95 ± 1.33	21	7
	Magnetic (manual)K8-peptide	44 ± 6	0.22 ± 0.05	210 ± 47	901	14
	Magnetic (manual)K16-peptide	163 ± 13	1.52 ± 0.03	107 ± 11	459	52
	Magnetic (automated)K8-peptide	29 ± 0	0.24 ± 0.05	125 ± 28	536	9
	Magnetic (automated)K16-peptide	131 ± 5	1.22 ± 0.21	110 ± 15	472	42

The numbers in “purification fold” and “yield” columns show the increase in EVs purity and the percentage recovery of EVs from the MCF7 supernatant, respectively. Each experiment was performed with three technical replicates.

^a^This data corresponds to EVs and proteins of MCF7 supernatant without purification (raw material).

### Automated purification of EVs

The Magtration Technology, which allows for the separation and resuspension of magnetic beads within a disposable pipette tip, has been developed by Precision System Science Co., Ltd.^32^. Magnetic beads are captured on the inner wall of the disposable tip and then resuspended in a new buffer. All processes, including the suspension of magnetic beads, separation of the magnetic beads, and resuspension of the magnetic beads in a new buffer, are performed automatically within a disposable tip.

**Figure 7:**
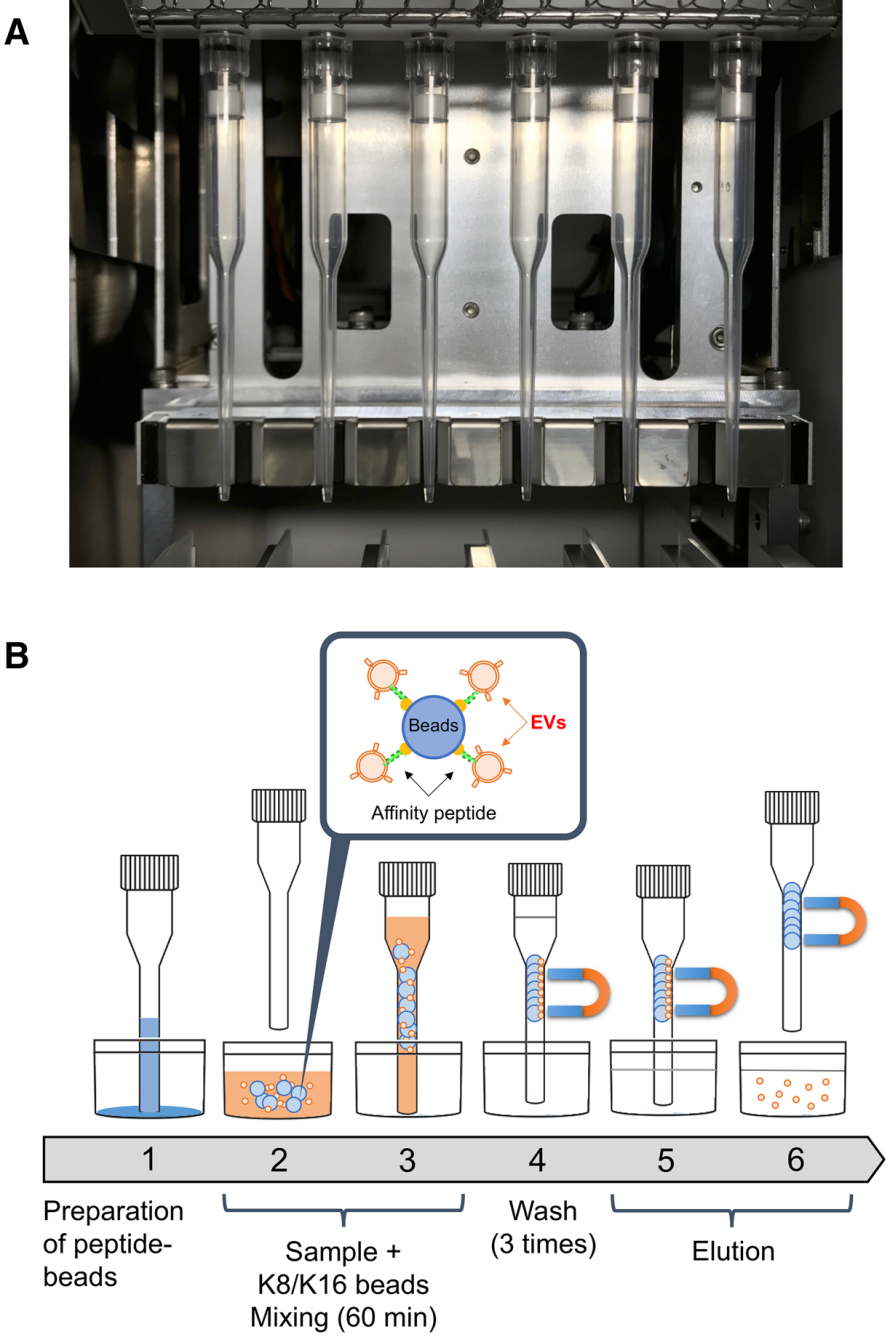
Schematic representation of the automated system used for EV purification. (**A**) Overview of tips in the pre-existing bench top instrument for magnetic separation. (**B**) Sequential scheme used for automated EV purification.

A schematic representation of our automated system for EV purification using peptide-immobilized magnetic beads is shown in Fig. 7. The purification was software-controlled to allow for the mixing of the beads, their washing, and the dissociation of EVs from the beads. Initially, we found that the yield was decreased to approximately 5% probably due to non-specific adsorption of EVs onto the inner wall of the disposable tip (Fig. 7A). To prevent this, we increased the concentration of Tween 20 in the binding and washing buffers by fivefold. As a result, the yields of EVs purified from the MCF7 culture supernatants using K8- and K16-peptide-immobilized magnetic beads were restored to 9% and 42%, respectively (Table 1). These results were similar to those obtained using the manual method. The purities of EVs automatically isolated using the K8- and K16-peptide-immobilized beads were 25- and 22-fold higher, respectively, than those purified by the differential ultracentrifugation method (Table 1).

## Discussion

To investigate the physicochemical and biochemical properties of EVs, a number of techniques, including ultracentrifugation-, size-, and immunoaffinity capture-based methods, have been developed for EV isolation. The results of EV isolation using multiple techniques suggest that the functional outcomes of EVs are technique-dependent^33^. The amount of EVs collected using the standard ultracentrifugation method is not consistent because the small, fragile EV pellets present after centrifugation can easily be lost during the decantation step^20,34^, and this further depends on the rotor type (fixed angle or swinging bucket) and even on the angle of the rotor^35^. The low reproducibility and low inter-rater reliability in the isolation/purification of EVs has a substantial impact on the data generated and on the potential clinical utility of the purified EVs.

Here, we tested lysine peptides that are able to bind to numerous different phospholipids in order to develop an EV magnetic separation method. Following optimization of the number of lysine residues in the peptides, we explored a range of different elution conditions that allowed for the gentle release of EVs from the peptides. Finally we developed an automated EV purification method using a pre-existing bench top instrument for magnetic separation. Just before the submission of our work, Gori et al. reported the development of membrane curvature-sensing peptides that allowed for the capture of EVs on a silicon chip and were able to quantitate these EVs using a fluorescently-labeled antibody against tetraspanin^36^. The peptides they reported are amphipathic and strongly bind to EVs through both electrostatic and hydrophobic interactions, and so can only be used for analytical purpose at the moment. In contrast, our peptides bind to EVs reversibly and thus can also be used for the purification of EVs. An important finding in our study was that the bound EVs could be dissociated from the lysine peptides under mild conditions.

Purity and yield showed a trade-off relationship when K8- and K16-peptides were used (Table 1). The higher yield of the K16-peptide is probably due to the approximately 10-fold higher affinity of the K16-peptide than the K8-peptide for phospholipids (Fig. 4). The K16-peptide might also capture other free (non-EV) proteins through increased electrostatic interactions, resulting in a lower purity of the EVs. However, it should be noted that based on CD9 levels alone, the binding and dissociation efficiencies (Fig. 3) suggested that there should be no difference in the purification between the K8- and K16-peptide-immobilized magnetic beads. Unexpectedly, the purification yield increased (14–52%, 3.7-fold) when the EVs were quantitated using a CD9/CD63 sandwich ELISA kit (Table 1). It is plausible that the K16-peptide-immobilized beads might efficiently isolate CD9/CD63-double-positive EVs. However, further experiments are necessary to explore the differential potential of these peptides.

Although EVs are a promising source of cancer biomarkers, few EV biomarkers have been implemented in clinical practice. This is partly due to a lack of accurate isolation/purification methods as alluded to above^9^. Our automated EV purification platform, which is not labor intensive, is likely to contribute to the introduction of novel EV biomarkers into the clinic in the near future. However, there remain several issues to be solved. First, blood is a more complex fluid than cell culture supernatants and markedly heterogeneous. Our preliminary experiments indicate that there are small molecules that inhibit the binding of EVs to K-8 peptide-immobilized beads in blood serum. A preprocessing step using a gel-filtration spin-column that can remove small molecules from serum has been added to resolve this problem (“Supplementary Materials and Methods”). As a result, the yields of EVs purified from serum using K8- and K16-peptide-immobilized beads were 15% and 41%, respectively (Supplementary Table S1). The purities of EVs purified from serum using K8- and K16-peptide-immobilized beads were 640- and 193-fold, respectively (Supplementary Table S1). Although a preprocessing step is necessary, this method could become a promising procedure for utility in the clinic. Second, evidence implicating EVs in regenerative processes is rapidly accumulating and EV-mediated therapy is gathering increasing interest^10–16^. However, for a practical therapy to be developed, a large amount of EVs will be required per dose. Thus, using another type of benchtop instrument for magnetic separation that is able to handle five samples of maximum 20 mL (up to 100 mL) simultaneously (magLEAD 5bL, Precision System Science Co., Ltd.) and/or other purification systems, such as K8- or K16-peptide-immobilized liquid chromatography, may offer alternative solutions for this limitation.

## Methods

### Cell culture

The human breast cancer cell line MCF7 was obtained from the Japanese Collection of Research Bioresources Cell Bank (Osaka, Japan). Cells were cultured in Dulbecco’s Modified Eagle Medium (DMEM) (Thermo Fisher Scientific, Hanover Park, IL, USA), supplemented with 10% heat-inactivated fetal bovine serum (HyClone FBS; GE Healthcare Life Sciences, Logan, UT, USA), 100 units/mL penicillin, and 0.1 mg/mL streptomycin in a humidified incubator with 95% air and 5% CO_2_ at 37 °C. After 48 h (cells reached a confluency of about 80%), the media was changed to a serum-free synthetic medium (advanced DMEM, Thermo Fisher Scientific) followed by a 48-h incubation.

### Isolation of EVs by differential ultracentrifugation

The conditioned medium described above was collected and centrifuged at 2000 × *g* for 10 min and then at 10,000 × *g* for 30 min. The resulting supernatant was filtered through a 0.2-µm membrane to remove cells and cellular debris, as only EVs < 0.2 µm, representing exosomes and smaller MVs, were the focus of this study, and then stored as a culture supernatant at − 80 °C. To prepare EVs, the MCF7 culture supernatant was thawed and centrifuged at 100,000 × *g* for 70 min at 4 °C using an Optima TLX equipped with a TLA100.3 fixed-angle rotor (Beckman Coulter, Miami, FL, USA). The supernatant was discarded, and the pellet was resuspended in phosphate-buffered saline (PBS, pH 7.4). The mixture was re-centrifuged at 100,000 × *g* for 70 min at 4 °C and the pellet was resuspended in PBS.

### Preparation of affinity peptide-immobilized magnetic beads

The N-terminal biotinylated peptide comprising three GGGS linkers and 8 lysine residues (K8-peptide: GGGSGGGSGGGSKKKKKKKK) was synthesized by Eurofin Genomics (Tokyo, Japan). Similarly, peptides comprising 4 lysine residues (K4-peptide: GGGSGGGSGGGSKKKK) and 16 lysine residues (K16-peptide: GGGSGGGSKKKKKKKKKKKKKKKK) were synthesized by Eurofin Genomics. Twenty microliters of streptavidin magnetic beads (GE Healthcare Life Sciences) were recovered using a magnetic stand and washed with 200 µL of BW buffer (20 mM Bis–Tris, pH 6.0; 150 mM NaCl; 0.005% Tween 20) and resuspended in 180 µL of BW buffer. To immobilize the biotinylated lysine-rich affinity peptides onto the streptavidin magnetic beads, 20 µL of biotinylated lysine-rich affinity peptides (100 µM) were added to the suspended beads and mixed using a microtube mixer (MT-400; Tomy, Tokyo, Japan) for 20 min. The beads were then washed three times with 200 µL of BW buffer, and finally resuspended in 50 µL of BW buffer and stored at 4 °C until use.

### Detection of the EV-CD9 marker bound to affinity peptide-immobilized magnetic beads

Two hundred microliters of the MCF7 culture supernatant was mixed with 20 µL of BT buffer (220 mM Bis–Tris, pH 6.0; 0.055% Tween 20) and 20 µL of the affinity peptide-immobilized magnetic beads solution followed by mixing using a microtube mixer MT-400 for 30 min. The beads were washed three times with 300 µL of BW buffer. Non-specific binding sites were blocked by treating the beads with 100 µL of TBS-T (Tris-buffered saline purchased from Nacalai Tesque, Kyoto, Japan and 0.05% Tween 20) containing 20 mg/mL fatty-acid-free bovine serum albumin (BSA; Wako, Osaka, Japan) for 20 min. Then, 100 µL of anti-CD9 antibody (Cosmo Bio Co., Ltd, Tokyo, Japan; diluted 1/1000 with TBS-T buffer containing BSA) was added and incubated for 30 min. After the beads were washed two times with TBS-T, 100 µL of horse-radish peroxidase (HRP)-conjugated anti-mouse IgG antibody (Abcam, Cambridge, UK; diluted 1/50,000 with TBS-T buffer containing BSA) was added followed by incubation for 30 min. After the beads were washed three times with TBS-T, the HRP activity was detected using a luminescent substrate with a microplate reader (Wallac 1420 ARVO MX; Perkin-Elmer, Boston, MA, USA).

### Manual purification of EVs using affinity peptide-immobilized magnetic beads

To isolate EVs, 500 µL of the MCF7 culture supernatant was mixed with 50 µL of BT buffer and 50 µL of affinity peptide-immobilized magnetic beads, followed by rotary mixing for 1 h. The beads were separated using a magnet stand, and then washed three times with 1 mL of BW buffer. After washing, the beads were mixed with 50 µL of elution buffer (20 mM Tris–HCl, pH 7.5; 500 mM NaCl) and incubated for 10 min. The beads were separated using the magnet stand and the supernatant (EV fraction) was transferred to a new 1.5-mL tube.

### Automated purification of EVs using Magtration Technology

The magLEAD 6gC (Precision System Science Co., Ltd., Matsudo, Japan) that can parallelly process a maximum of six samples was originally developed for DNA purification using the automated Magtration Technology. Before starting purification of EVs, 500 µL of the MCF7 culture supernatant was mixed with 50 µL of BT-2 buffer (220 mM Bis–Tris, pH 6.0; 0.275% Tween 20). The culture supernatant samples were then transferred to the magLEAD 6gC in which 20 µL of biotinylated K8- or K16-peptide, 50 µL of magnetic beads, 800 µL of BW-2 buffer (20 mM Bis–Tris, pH 6.0; 150 mM NaCl; 0.025% Tween 20), disposable tips, 50 µL of elution buffer, and tubes had been preset. The automated EV purification method involved the following steps: (1) mixing of the affinity peptide and streptavidin magnetic beads for 20 min; (2) mixing of the supernatant sample and the affinity peptide-immobilized magnetic beads for 60 min; (3) washing of the magnetic beads with BW-2 buffer; (4) elution of the EVs from the magnetic beads with elution buffer; and (5) the transfer of EVs into new tubes.

### Western blotting

The purified EV fractions were mixed with 4 × SDS sample buffer (0.2 M Tris–HCl, pH 6.8; 8% SDS; 40% glycerol; 0.4% bromophenol blue) and incubated at 37 °C for 30 min prior to electrophoresis (for CD9 and CD63 detection). The purified EV fractions were mixed with 4 × SDS sample buffer containing 2-mercaptoethanol and incubated at 65 °C for 5 min prior to electrophoresis (for TSG101 and Calnexin detection). EVs bound to the K8-peptide magnetic beads were eluted and lysed with 1 × SDS sample buffer (50 mM Tris–HCl, pH 6.8; 2% SDS; 10% glycerol; 0.1% bromophenol blue). MCF7 cells were disrupted by ultrasonication (Branson, Danbury, CT, USA) and used as a cell lysate. These samples were subjected to SDS–polyacrylamide gel electrophoresis (PAGE) and immunoblotting was performed using an anti-human CD9 antibody (1:2000, clone 12A12, Cosmo Bio Co., Ltd), an anti-human CD63 antibody (1:2000, clone 3–13, Wako), an anti-human TSG101 antibody (1:1000, ab30871, Abcam), or an anti-human Calnexin antibody (1:2000, ab22595, Abcam). HRP-conjugated anti-mouse IgG antibody (1:10,000, Abcam) and HRP-conjugated anti-rabbit IgG antibody (1:10,000, Abcam) were used as the secondary antibodies as appropriate. Immunoreactive proteins were detected and analyzed using a EzWestBlue substrate (ATTO, Tokyo, Japan).

### Phospholipid binding experiments

Phospholipid binding experiments were performed as previously described^37^. Briefly, phospholipids (100 µL, 0.75 µg/mL) suspended in methanol were added to 96-well F16 Black Polysorp Fluoronunc Plates (Thermo Fisher Scientific) and air-dried. Non-specific binding sites were blocked by treating the wells with a PBS-blocking solution containing 10 mg/mL fatty-acid-free BSA (Wako). The biotinylated K8-peptide was diluted to 100 nM with the PBS-blocking solution, and then added to the wells, followed by incubation for 30 min. Unbound K8-peptides were removed by washing the wells with 100 µL of PBS containing 0.05% Tween 20. Then, HRP-conjugated streptavidin (Thermo Fischer Scientific), diluted 10,000-fold with the PBS-blocking solution containing 0.05% Tween 20, was added to the wells, followed by incubation for 30 min. After the wells were washed with PBS containing 0.05% Tween 20, HRP activity was assessed by adding a luminescent peroxidase substrate and measuring the resulting luminescence on a microplate reader (Wallac 1420 ARVO MX). The luminescence data were fitted to the Langmuir isotherm to determine the equilibrium dissociation constant (Kd). The Kd was obtained by fitting the luminescence intensity obtained from specific binding at each concentration of peptide using the equation:${\text{Y}} = {{{\text{B}}_{{\max}} \cdot {\text{X}}} \mathord{\left/ {\vphantom {{{\text{B}}_{{\max}} \cdot {\text{X}}} {\left( {{\text{Kd}} + {\text{X}}} \right)}}} \right. \kern-\nulldelimiterspace} {\left( {{\text{Kd}} + {\text{X}}} \right)}},$ where Y represents the luminescence intensity, X represents the peptide concentration, and B_max_ represents the maximum luminescence intensity (maximum binding capacity of the peptide to PS).

For the competitive assay, the wells were blocked with Tris–HCl buffer (10 mM Tris, pH 7.4; 150 mM NaCl) containing 10 mg/mL BSA. The biotinylated K8-peptide (100 nM) was added to the wells in the presence of 30 mM sodium phosphate, 10 mM sodium triphosphate, or 2 mM sodium polyphosphate (15 phosphate residues) solution, followed by incubation for 30 min. After washing the wells with Tris–HCl buffer containing 0.05% Tween 20, HRP-conjugated streptavidin, diluted 10,000-fold with Tris–HCl buffer containing 10 mg/mL BSA and 0.05% Tween 20, was added to the wells, and incubated for 30 min. After the wells were washed with Tris–HCl buffer containing 0.05% Tween 20, HRP activity was determined as described above.

### Quantification of EVs using CD9/CD63 ELISA

The EV samples were diluted to the appropriate concentrations and assayed following the instructions provided along with the CD9/CD63 ELISA kit (Cosmo Bio Co., Ltd). A calibration curve was developed using different amounts of the CD9-CD63 fusion protein included in this kit. EV concentrations in the samples were calculated as equivalent to the CD9-CD63 fusion protein. Total protein concentration was measured using the Micro-BCA protein assay (Thermo Fisher Scientific).

### Scanning electron microscopy

Purified EVs, which had been dialyzed against ultrapure water, were placed onto the carbon tape (Nisshin EM Co., Ltd, Tokyo, Japan) and fixed with 2.5% glutaraldehyde. After washing with ultrapure water, the samples were dried overnight at 37 °C. Carbon-shadow coating was performed using CADE-E (Meiwafosis, Osaka, Japan). The samples were then observed using a field-emission scanning electron microscope (FE-SEM, Sigma VP; Carl Zeiss Microscopy GmbH, Jena, Germany).

### Transmission electron microscopy

Purified EVs were added onto glow-discharged carbon-film grids. The grid was dried with filter paper and stained with 2% uranyl acetate in double distilled water for 10 s. After the stain was completed, the grid was washed with distilled water and blotted dry with filter paper. The grid was air dried and visualized under the Hitachi H-7600 TEM (Hitachi, Japan) operating at 100 kV.

### Chemicals

l-Phosphatidylcholine and l-phosphatidylinositol were purchased from Nacalai Tesque (Kyoto, Japan). l-Phosphatidylethanolamine and l-phosphatidyl-l-serine were purchased from Sigma-Aldrich (St. Louis, MO, USA). Other chemicals were purchased from Wako.

### Statistical analysis

The data are presented as means of three independent experiments. The standard deviations of each set of experiments are represented in figures (as bars) and in the table. Statistical analysis was carried out using Student’s *t*-test. A value of p < 0.05 was considered significant.

## Supplementary information

Supplementary Information.

## Data Availability

The datasets generated during and/or analyzed during the current study are available from the corresponding author upon reasonable request.
