# Supplementary material for: Application of peptides with an affinity for phospholipid membranes during the automated purification of extracellular vesicles
Source: Sci Rep. 2020 Oct 30;10:18718. doi: 10.1038/s41598-020-75561-0 (PMC7603496; doi:10.1038/s41598-020-75561-0)
Supplement: Supplementary file 1 — Supplementary Information. [file 41598_2020_75561_MOESM1_ESM.docx]

Supplementary Materials for

**Application of peptides with an affinity for phospholipid membranes during the automated purification of extracellular vesicles**

Takenori Ishida^1^, Takuma Hashimoto^1^, Kanako Masaki^1^, Hisakage Funabashi^1^, Ryuichi Hirota^1^, Takeshi Ikeda^1^, Hideji Tajima^2^, Akio Kuroda^1*^

1, Unit of Biotechnology, Graduate School of Integrated Sciences for Life, Hiroshima University, 1-3-1 Kagamiyama, Higashi-Hiroshima, Hiroshima 739-8530, Japan

2, Precision System Science Co., Ltd., 88 Kamihongo, Matsudo, Chiba 271-0064, Japan

*Corresponding authors：Akio Kuroda, akuroda@hiroshima-u.ac.jp

**Supplementary Materials and Methods**

*EV purification from human blood serum.* As a preprocessing step, 100 µL of human blood serum (Biowest, France) was passed through a MicroSpin S-400 HR Column (GE Healthcare Life Sciences) and diluted with 400 µL of BW-3 buffer (100 mM MES, pH5.5; 150 mM NaCl; 0.005% Tween20). For the preparation of magnetic beads, 60 µL of streptavidin magnetic beads (GE Healthcare Life Sciences) were recovered using a magnetic stand and washed with 600 μL of BW-3 buffer and resuspended in 540 μL of BW-3 buffer. Then, 60 μL of biotinylated K8- or K16-peptide (100 μM) was added to the suspended beads and mixed using a microtube mixer (MT-400; Tomy) for 20 min. The beads were then washed three times with 600 μL BW-3 buffer, and finally resuspended in 50 μL BW-3 buffer. To isolate EVs, 500 µL of the preprocessed blood serum was mixed with 50 µL affinity peptide-immobilized magnetic beads, followed by rotary mixing for 1 h. The beads were separated using a magnet stand, and then washed three times with 1 mL BW-3 buffer. After washing, the beads were mixed with 50 µL of elution buffer-2 (50 mM Tris-HCl, pH 8.0; 500 mM NaCl) and incubated for 10 min. The beads were separated using the magnet stand and the supernatant was transferred to a new 1.5-mL tube. This elution was repeated two times. These two supernatants were mixed together and stocked as an EVs fraction. The EV fraction was assayed with the CD9/CD63 ELISA kit (Cosmo Bio Co., Ltd). Total protein concentration was measured using the Micro-BCA protein assay (Thermo Fisher Scientific).

**Table S1, EV purification from human blood serum.**

| Sample | Purification method | CD9/CD63 amount in EVs (pg) | Total protein (μg) | Purity  (CD9/CD63 amount  /total protein) | Purification fold | Yield (%) |
| --- | --- | --- | --- | --- | --- | --- |
| Human blood serum |  | 103 ± 1.5 | 7980 ± 626 | 0.013 ± 0.001 | 1 | 100 |
|  | Microspin column | 71.7 ± 2.9 | 1050 ± 148 | 0.068 ± 0.0087 | 5.2 | 70 |
|  | Microspin column + Magnetic (manual)K8-peptide | 15.8 ± 0.8 | 1.9 ± 0.2 | 8.32 ± 0.48 | 640 | 15 |
|  | Microspin column + Magnetic (manual)K16-peptide | 42.5 ± 0.8 | 16.9 ± 0.2 | 2.51 ± 0.05 | 193 | 41 |

The numbers in “purification fold” and “yield” columns show the increase in EVs purity and the percentage recovery of EVs from the serum, respectively. Each experiment was performed in three technical replicates.


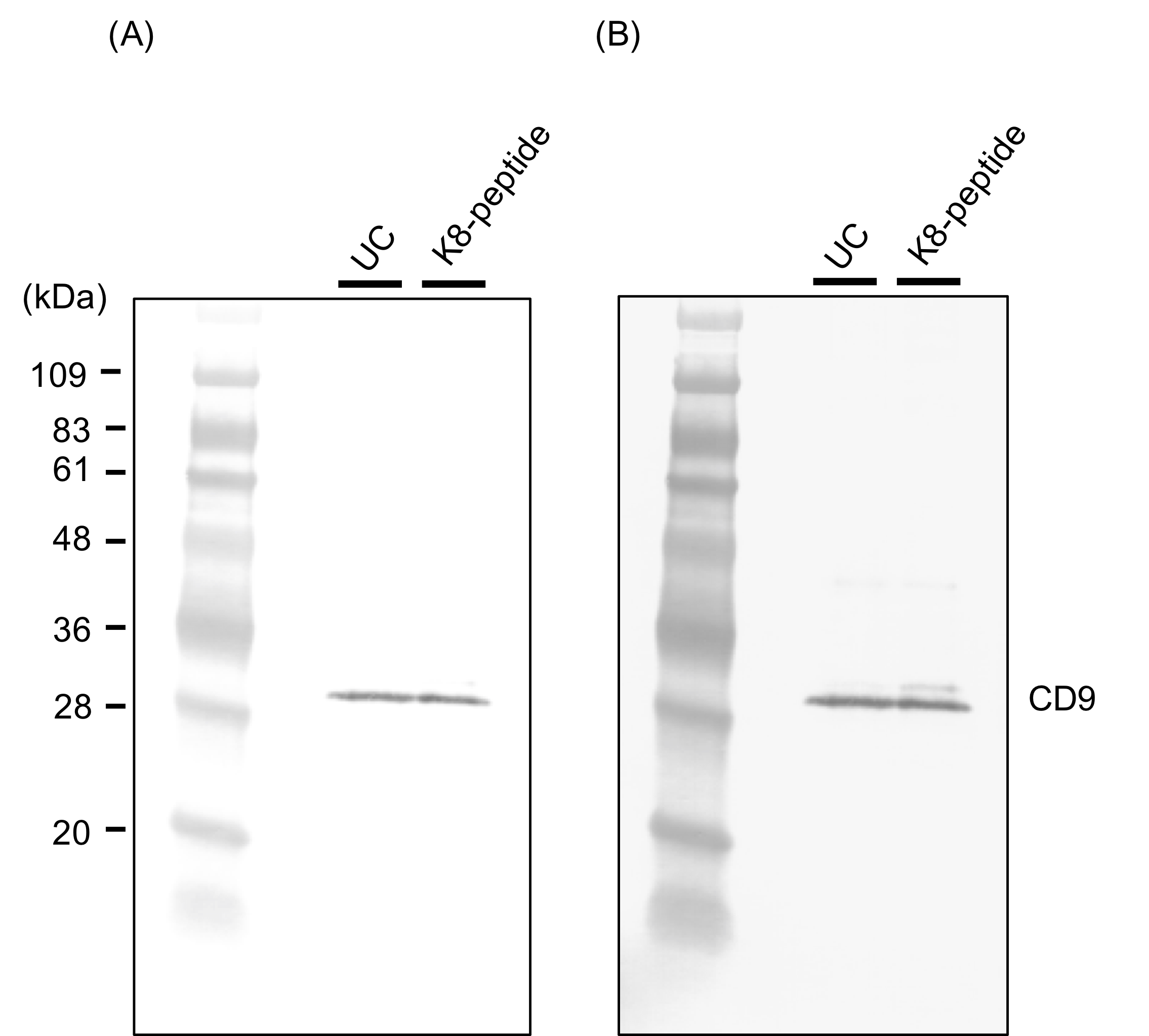


**Figure S1.** Full western blots of the EVs bound to K8-peptide magnetic beads with a CD9 antibody (A and B different exposition times). Cropped version is presented in Figure 1.


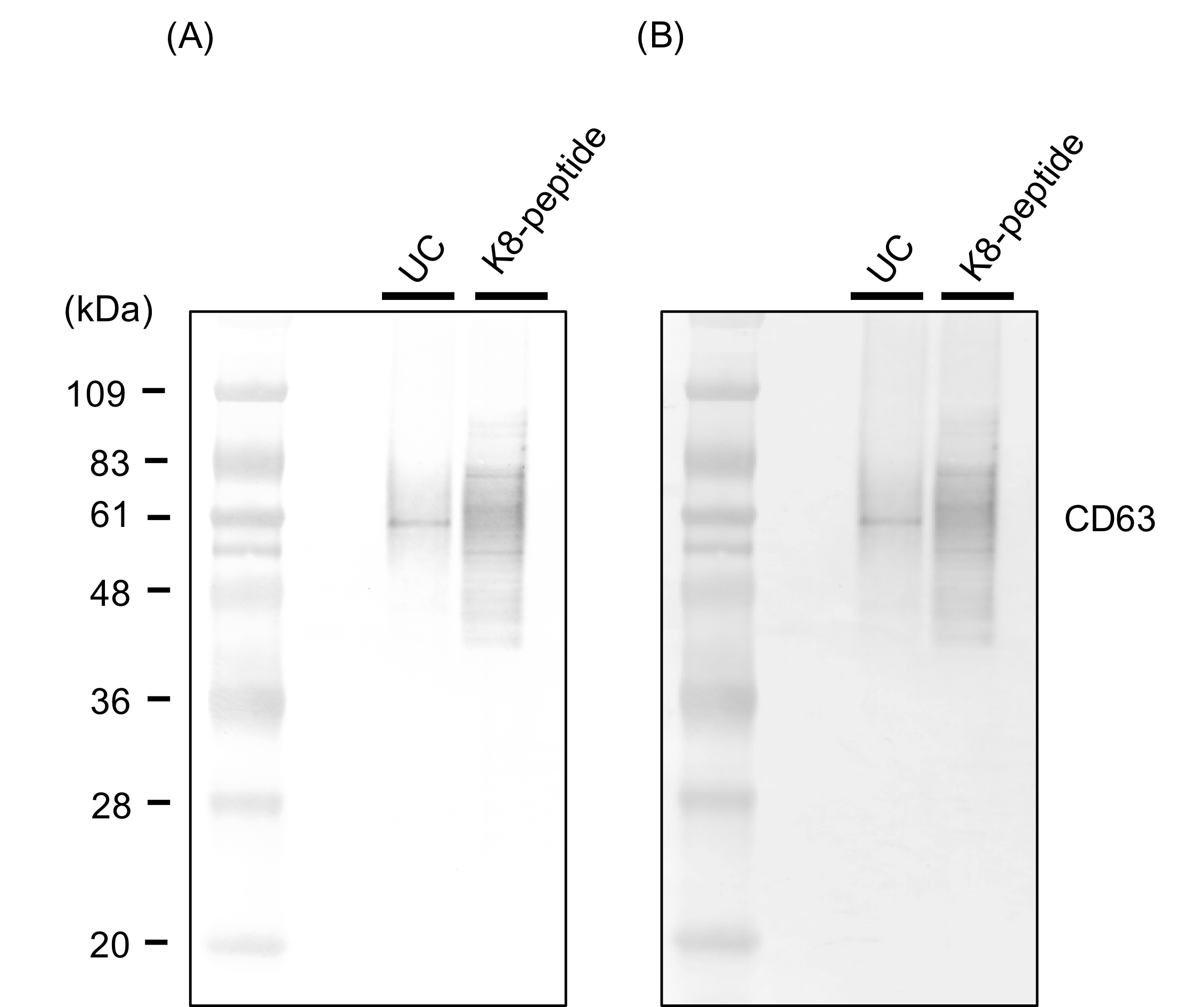


**Figure S2.** Full western blots of the EVs bound to K8-peptide magnetic beads with a CD63 antibody (A and B different exposition times). Cropped version is presented in Figure 1.


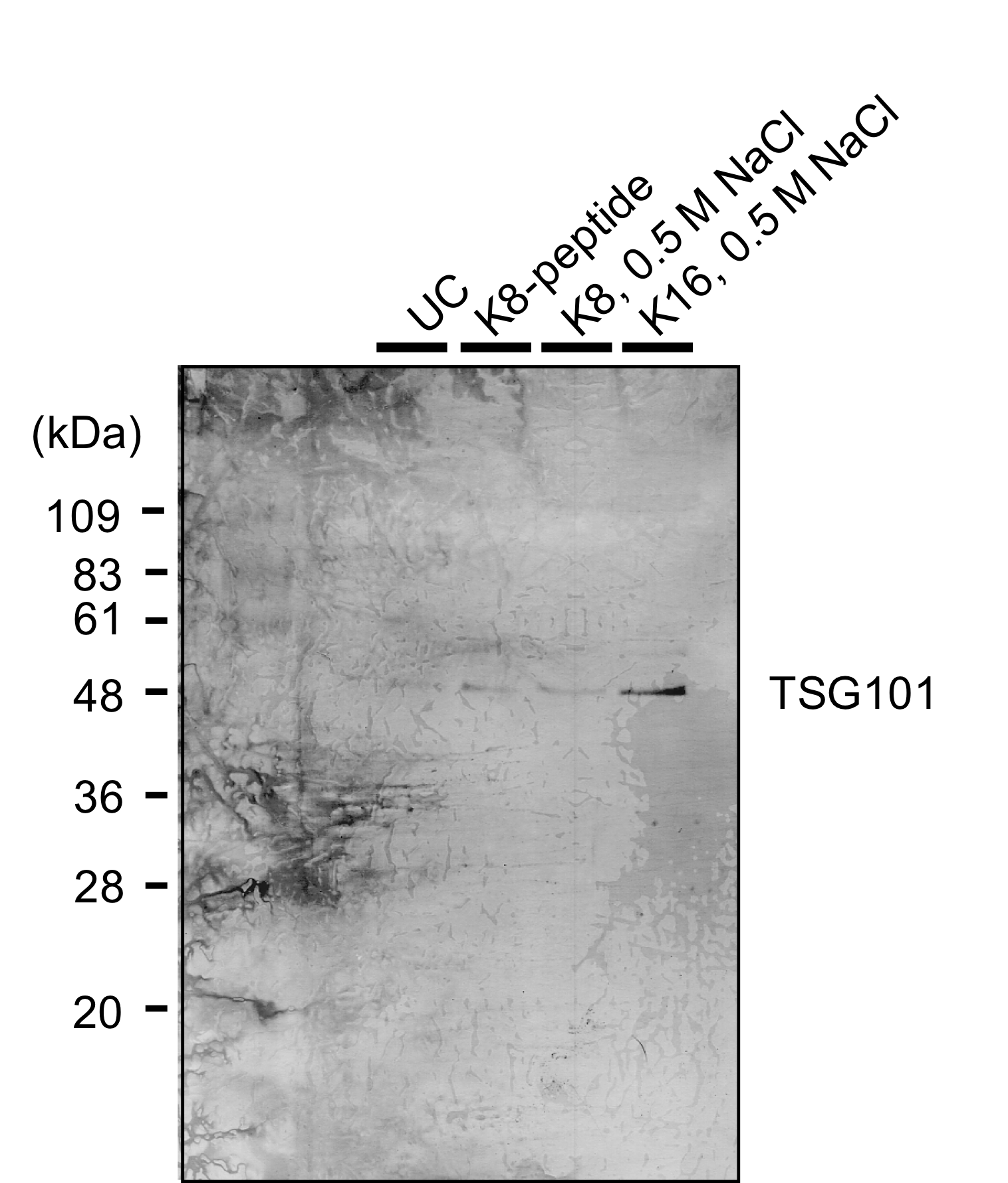


**Figure S3.** Full western blots of the EVs bound to K8-peptide magnetic beads with a TSG101 antibody. Cropped version is presented in Figure 1 and Figure 5.


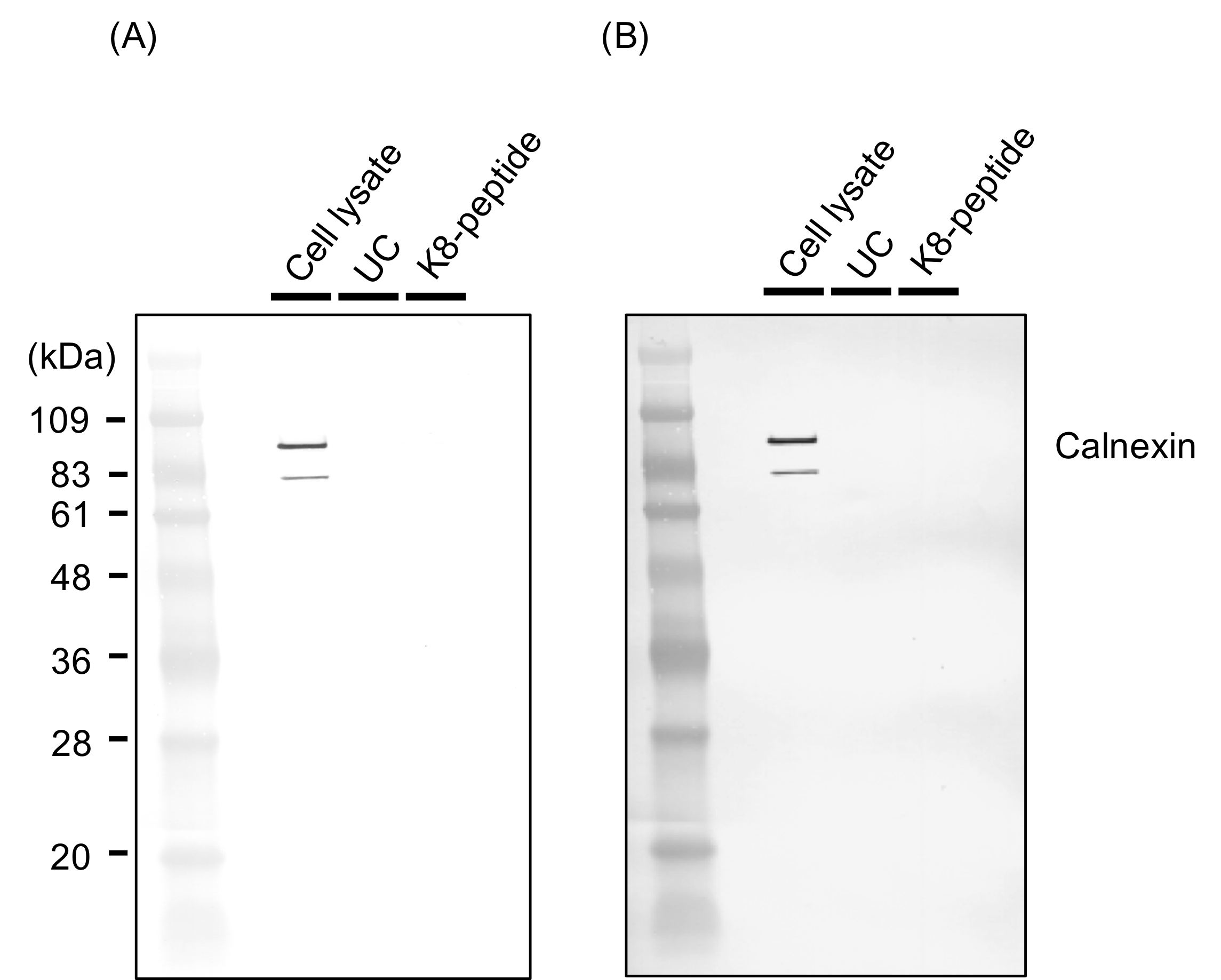


**Figure S4.** Full western blots of the EVs bound to K8-peptide magnetic beads with a Calnexin antibody (A and B different exposition times). Cropped version is presented in Figure 1.


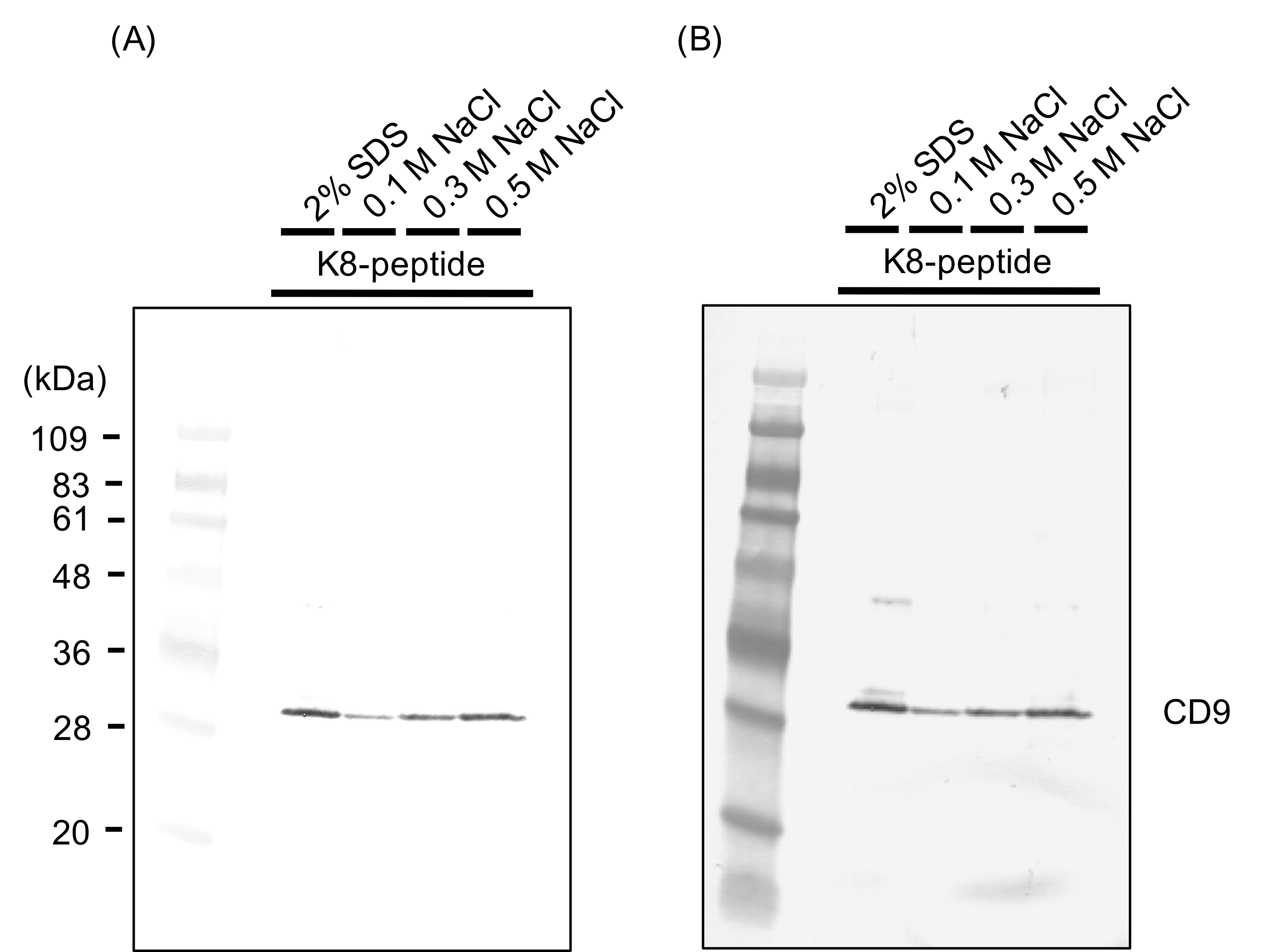


**Figure S5.** Full western blots of the EVs dissociated from K8-peptide magnetic beads with a CD9 antibody (A and B different exposition times). Cropped version is presented in Figure 5.


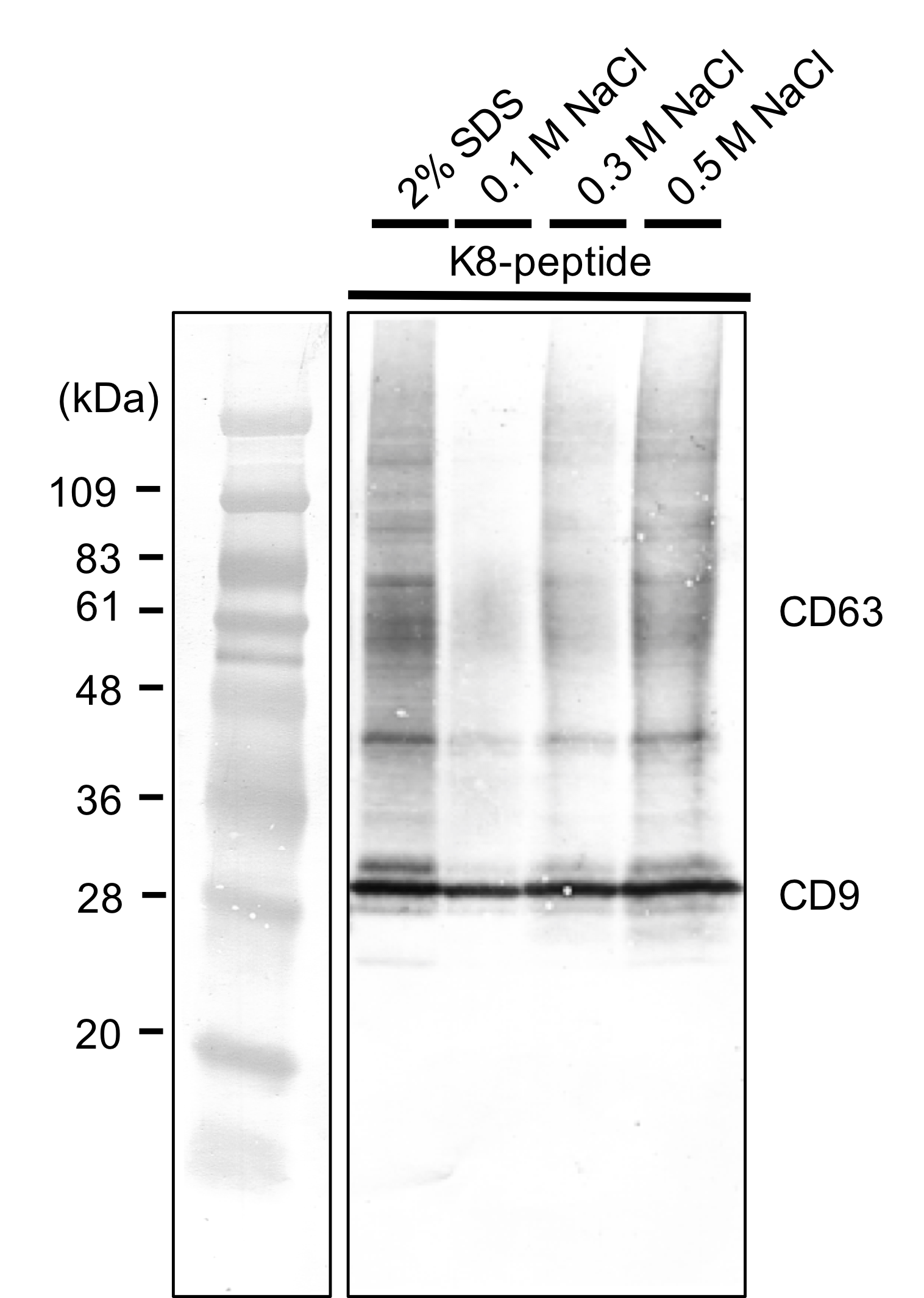


**Figure S6.** Full western blots of the EVs dissociated from K8-peptide magnetic beads with CD9 and CD63 antibodies. Cropped version (for CD63 probing) is presented in Figure 5.
